# Myocardial Injury as a Harbinger of Multi-organ Failure in Septic Shock: A Comprehensive Review

**DOI:** 10.7759/cureus.55021

**Published:** 2024-02-27

**Authors:** Amol Singam

**Affiliations:** 1 Critical Care Medicine, Jawaharlal Nehru Medical College, Datta Meghe Institute of Higher Education and Research, Wardha, IND

**Keywords:** therapeutic interventions, diagnostic approaches, prognosis, multi-organ failure, septic shock, myocardial injury

## Abstract

Myocardial injury in the context of septic shock presents a multifaceted challenge in critical care medicine with implications for patient prognosis and therapeutic strategies. This comprehensive review explores the mechanisms, diagnostic approaches, and clinical implications of myocardial injury as a harbinger of multi-organ failure in septic shock. We delineate the pathophysiological processes underlying myocardial injury, including inflammation, oxidative stress, and microcirculatory dysfunction, and discuss the diagnostic utility of cardiac biomarkers and imaging modalities in identifying myocardial injury. Furthermore, we elucidate the prognostic significance of myocardial injury and its implications for patient management, highlighting the need for tailored therapeutic interventions to mitigate its adverse effects. Future research and clinical practice directions are also discussed, emphasizing the importance of personalized medicine approaches and multidisciplinary collaboration in optimizing outcomes for patients with septic shock-associated myocardial injury. This review aims to provide a comprehensive understanding of myocardial injury in septic shock and inform strategies for improving patient care in this challenging clinical scenario.

## Introduction and background

Septic shock is a life-threatening condition characterized by a dysregulated host response to infection, resulting in profound circulatory dysfunction and cellular damage. It represents the most severe manifestation of sepsis, with mortality rates exceeding 40% despite advances in medical care [1]. Shock myocardial injury, often occurring in the setting of septic shock, involves damage to the heart muscle due to various pathological processes, including inflammation, oxidative stress, and microcirculatory dysfunction. It manifests as elevated cardiac biomarkers such as troponins and is associated with adverse clinical outcomes [2].

Recognizing myocardial injury as a harbinger of multi-organ failure is crucial for timely intervention and prognostication in patients with septic shock. The heart's role in orchestrating systemic responses to infection underscores its significance as both a target and a predictor of organ dysfunction in this critical condition [3]. This comprehensive review aims to elucidate the mechanisms underlying myocardial injury in septic shock, explore its diagnostic and prognostic implications, and discuss therapeutic strategies to mitigate its impact on patient outcomes. By synthesizing current evidence and highlighting gaps in knowledge, this review seeks to inform clinical practice and inspire further research in this vital area of critical care medicine.

## Review

Myocardial injury in septic shock

Definition and Classification of Myocardial Injury

Myocardial injury can arise from a range of both cardiac and non-cardiac conditions, resulting in either acute or chronic myocardial damage. The universal definition of myocardial infarction delineates a classification based on etiology. Type 1 is myocardial injury, which is different from myocardial infarction. This classification acknowledges the central role of coronary artery disease in type 1 myocardial infarction caused by plaque rupture, while type 2 encompasses myocardial injury due to other factors [4]. Myocardial injury may stem from a mismatch in myocardial oxygen supply-demand or the context of alternative acute illnesses such as sepsis or viral myocarditis. The specific mechanism of myocardial injury dictates the necessity for cardiac or coronary investigations and treatment strategies. Identifying and categorizing patients with acute or chronic myocardial injury is pivotal for directing further investigations and interventions [4]. Within the realm of sepsis, myocardial injury emerges as a frequent complication independently linked to early mortality. The pathophysiology of sepsis-induced myocardial injury (SIMI) involves a myriad of mechanisms, including cardiac dysfunction, yielding significant morbidity and mortality rates. Notably, sepsis-induced myocardial dysfunction is reversible and often characterized by left ventricular depression, with most patients experiencing full recovery over several days [5-7]. Proper classification and evaluation of myocardial injury, whether in the context of acute coronary syndrome (ACS) or sepsis, are imperative for guiding clinical management and enhancing patient outcomes. Further research is warranted to deepen our understanding of myocardial injury mechanisms across various clinical conditions and to devise more efficacious therapeutic approaches.

Incidence and Clinical Significance

The incidence of septic cardiomyopathy (SCM) exhibits a wide variation, with reported prevalence spanning from 10% to 70% [8,9]. SCM is characterized by reversible myocardial dysfunction triggered by sepsis, resulting in diminished myocardial contractility and left ventricular ejection fraction (LVEF), typically reverting to baseline function within seven to 10 days [8]. Despite ongoing research, the clinical significance of myocardial injury in sepsis remains a subject of investigation, with the specific implications and long-term effects of SCM still being clarified [9]. Although the precise clinical features and outcomes of SCM are not fully delineated, it is acknowledged as a severe condition that can significantly impact the prognosis of sepsis or septic shock [7]. Further studies are warranted to define SCM's epidemiology and clinical significance more precisely, alongside the development of standardized diagnostic and management protocols for this condition [9].

Mechanisms of Myocardial Injury in Septic Shock

Inflammatory response: The inflammatory response in septic shock is a multifaceted process characterized by activating leukocytes, endothelial cells, and the coagulation system. In cases of severe sepsis, a systemic inflammatory cascade is triggered by the release of bacteria, their toxins, and various inflammatory mediators. This cascade induces a hyperinflammatory state and compromises innate immune functions, culminating in septic shock and multi-organ failure [10-12]. The intensity of the inflammatory response in sepsis varies, ranging from moderate to intense. Both human and rodent studies have demonstrated that the development of systemic inflammatory response syndrome in sepsis is associated with a disruption of the redox balance, as well as heightened inflammation and impaired innate immune functions [11,12]. Throughout the progression, resolution, and long-term outcome of sepsis, the immune system's role is marked by a persistent and simultaneous inflammatory and anti-inflammatory state driven by dysfunctional innate and adaptive immune systems [13]. While the inflammatory response in sepsis alerts the immune system to the presence of infection, it becomes dysregulated during sepsis, resulting in a hyperinflammatory state and immune suppression [12].

Microcirculatory dysfunction: Microcirculatory dysfunction emerges as a prevalent characteristic of sepsis, exerting a pivotal role in the disease's pathophysiology. These changes involve the impairment of nitric oxide-induced arteriolar vasodilation and heightened perfusion heterogeneity due to inappropriate vasoconstriction, ultimately resulting in diminished oxygen delivery, tissue hypoxia, and organ dysfunction [14-16]. Notably, microcirculatory dysfunction manifests early in the course of sepsis, with the severity of derangement correlating with disease severity and prognosis in ICU patients [15-16]. Despite its significance, monitoring the microcirculation poses challenges in clinical practice. It often relies on macro-hemodynamic variables such as arterial pressure, cardiac output, heart rate, and efforts to restore organ perfusion [15]. However, studies indicate that improvements in microcirculatory function following early resuscitation are associated with reduced mortality rates [15]. Consequently, restoring microcirculatory function emerges as a promising therapeutic target for resuscitation efforts, although further data are warranted to fully elucidate its potential benefits [15-16]. Recognizing the critical role of microcirculation in sepsis underscores the importance of comprehending the processes underlying microvascular dysfunction, guiding clinicians away from solely chasing systemic hemodynamic parameters and towards more targeted resuscitation strategies [16].

Cellular and metabolic alterations: Sepsis precipitates significant metabolic disruptions, prominently characterized by dysfunction and injury to mitochondria, and is identified as a primary instigator of cellular metabolic dysregulation. These alterations affect the metabolism of all macronutrients, fostering intensified glycolysis, heightened lactate formation, and elevated lipolysis in adipose tissue, among other metabolic shifts [17]. The hyperinflammatory state induced by sepsis exacerbates energy deficits, prompting adaptations in cellular metabolism and necessitating a redirection of energy expenditure [18]. Mitochondrial dysfunction assumes a central role in the sepsis pathophysiology. It is closely linked to patient outcomes, contributing to the impairment of metabolic function and the reduction of mitochondrial respiratory capacity [18]. The intricacies of cellular and metabolic dysfunction in sepsis involve a complex interplay of interconnected pathways, encompassing carbohydrate and lipid metabolism alterations alongside the repercussions of mitochondrial dysfunction on cellular homeostasis [17-18].

Oxidative stress and mitochondrial dysfunction: Oxidative stress and mitochondrial dysfunction are intricately intertwined and implicated in the pathogenesis of various diseases. Mitochondria are a primary source of intracellular reactive oxygen species (ROS) and are particularly susceptible to oxidative stress. The excessive generation of ROS and the impairment of defensive antioxidant mechanisms can induce mitochondrial dysfunction, characterized by damage to the mitochondrial respiratory chain, membrane permeability perturbation, and cellular homeostasis disruption [19-21]. Consequently, this cascade may lead to the overproduction of free radicals, resulting in oxidative damage to crucial cellular components such as DNA, proteins, and lipids [19,20]. Notably, mitochondrial dysfunction and oxidative stress have been linked to a broad spectrum of disorders, spanning metabolic conditions, neurodegenerative diseases, and cardiac ailments [20-23]. Given the pivotal role of mitochondrial oxidative stress in disease pathogenesis, there has been growing interest in developing mitochondria-targeted antioxidant therapies as potential treatment modalities [21]. Therefore, elucidating the intricate relationship between oxidative stress and mitochondrial dysfunction represents a critical area of investigation with far-reaching implications for developing therapeutic interventions to mitigate disease progression and improve patient outcomes.

Diagnostic approaches

Biomarkers of Myocardial Injury

Troponins: Troponin is a pivotal cardiac biomarker extensively employed for diagnosing myocardial injury, spanning contexts like ACS and SCM. Comprising three subunits (troponin T, troponin C, and troponin I), troponin constitutes a protein complex crucial for cardiac muscle function. Troponin T and I isoforms, recognized as cardiac troponins (cTn), demonstrate high specificity and sensitivity to cardiac myocytes, rendering their detection in the bloodstream a particular indicator of cardiac damage. Troponin levels typically peak within 12-48 hours post-injury and remain elevated for four to 10 days. The sensitivity for detecting troponin T and I approaches 100% when sampled six to 12 hours after the onset of acute chest pain. Consequently, in the context of acute chest pain, to effectively rule out ACS, patients should undergo a repeat troponin sample six to 12 hours after the initial assessment. Notably, troponin is the most widely utilized biomarker for detecting cardiac injury, boasting unparalleled sensitivity. However, it's essential to acknowledge that other cardiac conditions, such as myocarditis, Tako-tsubo cardiomyopathy, or shock, can also significantly alter troponin levels. The interpretation of troponin results hinges heavily on the clinical context in which it is requested [24-26].

Natriuretic peptides: Natriuretic peptides, encompassing B-type natriuretic peptide (BNP) and N-terminal pro-B-type natriuretic peptide (NT-proBNP), stand as pivotal biomarkers widely utilized in diagnosing and prognosticating heart failure. These peptides play a fundamental role in managing patients with heart failure, offering invaluable assistance in diagnosis and prognostication and potentially serving as therapeutic targets. Among biomarkers studied and employed in heart failure, natriuretic peptides hold a prominent position, with both American and European guidelines recommending their measurement for heart failure diagnosis and risk stratification. Elevated levels of natriuretic peptides, particularly BNP, have demonstrated strong associations with adverse outcomes, including poorer prognosis, increased mortality, and a heightened risk of readmission. Furthermore, they aid in risk stratification and prognostic assessment in heart failure patients. Additionally, natriuretic peptides prove valuable in distinguishing heart failure from other conditions when clinical uncertainty arises. Despite the elevation of other biomarkers, such as troponins in heart failure, natriuretic peptides maintain their status as preferred biomarkers in clinical practice due to their specificity and clinical utility [27-30]. Thus, the essential role of natriuretic peptides in heart failure diagnosis and management, particularly BNP and NT-proBNP, underscores their critical importance in clinical practice.

Inflammatory markers: Inflammatory markers represent a subset of biomarkers utilized in diagnosing myocardial injury, reflecting the pivotal role of inflammation in its pathogenesis. Notably, several biomarkers of inflammation have been identified, including C-reactive protein (CRP), interleukin-6 (IL-6), and S100A9 [31-32]. CRP levels correlate with plaque rupture in patients experiencing acute myocardial infarction [32]. At the same time, IL-6, a pro-inflammatory cytokine released in response to tissue injury, has been consistently elevated in individuals with myocardial injury [31,33]. Additionally, S100A9, a protein released by neutrophils and monocytes, has been implicated in myocardial inflammation [32,34]. Despite their utility in diagnosing myocardial injury, these biomarkers lack specificity for SCM and can be elevated in other conditions as well [31-32]. Consequently, a comprehensive approach integrating various biomarkers and diagnostic modalities such as echocardiography, MRI, and ventriculography may be necessary to accurately diagnose SCM [31-34]. Thus, while inflammatory markers provide valuable insights into myocardial injury, their broader applicability underscores the importance of employing a multifaceted diagnostic strategy to differentiate SCM from other conditions.

Imaging Modalities

Echocardiography: Echocardiography is a non-invasive and dependable imaging modality for evaluating cardiac structure, function, and hemodynamics [35-37]. Widely regarded as the gold standard for diagnosing SCM [35-37], echocardiography utilizes high-frequency sound waves to generate heart images. It boasts accessibility, affordability, and a radiation-free nature [35-37]. Transthoracic echo, the most common type, involves transmitting ultrasound waves through the chest wall to capture images of the heart [36]. Stress echo, on the other hand, aids in assessing overall heart function and detecting abnormalities by visualizing changes in the heart's shape and dimensions during the cardiac cycle [36]. While operator-dependent, echocardiography's accuracy remains comparable to other imaging modalities [35-37].

Cardiac MRI: Cardiac MRI emerges as a non-invasive imaging tool employing a potent magnetic field, radio waves, and computer technology to produce detailed images of cardiac structures [37-39]. It utilizes various pulse sequences to assess cardiovascular disease, including morphological evaluation, cine imaging, blood flow measurement, and tissue composition assessment [38]. Cardiac MRI offers high-resolution images without ionizing radiation or iodinated contrast exposure, providing insights into ventricular and valvular function, cardiomyopathies, congenital heart disease, and cardiac tumors [38]. However, limited availability and contraindications in seriously injured patients or those with specific medical devices may restrict its use [39].

Computed tomography angiography (CTA): CTA integrates CT scanning with contrast material to visualize blood vessels and tissues throughout the body [40-41]. This non-invasive procedure aids in diagnosing and evaluating vascular diseases such as aneurysms, blockages, and vascular malformations in various anatomical regions [40-41]. CTA involves injecting contrast material into the blood vessels and capturing high-resolution CT images while the material circulates [40-41]. With minimal radiation exposure and lower complication risks compared to conventional angiography, CTA offers fast and effective vascular imaging [40-41]. Nonetheless, patients are advised to disclose any allergies to contrast material, and specific preparations may be necessary before the procedure [41]. Overall, the benefits of CTA outweigh the risks, making it a valuable diagnostic tool in vascular pathology assessment [41].

Clinical implications

Predictive Value of Myocardial Injury for Outcomes in Septic Shock

Research on the predictive value of myocardial injury for outcomes in septic shock has yielded significant insights. A study featured in the Annals of Intensive Care discovered that left ventricular systolic function parameters, such as mitral annular plane systolic excursion (MAPSE) and left ventricular longitudinal wall fractional shortening (LV-LWFS), were independently associated with myocardial injury in septic shock patients. These parameters, identified as indicative of myocardial injury upon ICU admission, warrant further exploration regarding their relationship with clinical outcomes [42]. In a separate review article, the intricate pathophysiology of SCM was underscored, implicating mechanisms such as bacterial endotoxin, inflammatory markers, ROS, and mitochondrial dysregulation. The review emphasized the imperative for additional investigation to ascertain the precise cause of myocardial dysfunction and develop innovative treatments [43]. Furthermore, a prospective observational study evaluated the prognostic value of high-sensitivity cardiac troponin I (hs-cTnI) in predicting outcomes among septic shock patients. The study revealed a notable prevalence of elevated hs-cTnI levels in these patients and demonstrated a significant association between elevated troponin and mortality [44]. The emerging predictive value of myocardial injury markers, including MAPSE, LV-LWFS, and hs-cTnI, presents promise for assessing outcomes in septic shock patients. Nevertheless, further research is imperative to comprehensively understand the interplay between myocardial injury and clinical outcomes in this patient population.

Impact on Treatment Strategies

The treatment landscape for SCM carries significant implications, with current approaches predominantly focusing on supportive care, infection control, and hemodynamic support [5,45-46]. There is growing interest in therapeutic strategies targeting mitochondrial dysfunction in sepsis as a potential avenue for intervention [45]. Management typically entails the administration of vasopressors such as noradrenaline, with vasopressin emerging as a potentially effective option for septic shock in some instances [47]. Additionally, inotropes like dobutamine and levosimendan have been explored, although further research is required to evaluate their efficacy [47]. In severe cases, mechanical support with intra-aortic balloon pumping (IABP) or extracorporeal membrane oxygenation (ECMO) has been considered, although it has not yet become standard practice [43,47]. Overall, treatment strategies for SCM represent an active area of research and clinical exploration, with a pressing need for enhanced understanding and the development of more effective therapeutic approaches.

Monitoring and Management Considerations

The management of SCM encompasses several key components, including etiological treatment, adapted fluid resuscitation, vasopressor use, and vigilant monitoring [8]. While the use of inotropes remains uncertain, heart rate control may be considered in select patients [8]. Echocardiography currently stands as the gold standard for diagnosing SCM, with the assessment of global longitudinal strain (GLS) potentially offering greater sensitivity and specificity than traditional measures such as LVEF [8]. Physicians should maintain a high index of suspicion for SCM in patients with sepsis-associated organ dysfunction, particularly those with septic shock requiring vasopressors [8]. Regular evaluation of myocardial function is recommended, with cautious fluid expansion and strict limitations [8]. Beta-blockers should be used judiciously and sparingly, with systolic dysfunction serving as a contraindication in most cases [8]. Despite current management strategies, further research is crucial to developing more effective therapeutic approaches for SCM [8].

Association with multi-organ failure

Relationship Between Myocardial Injury and Organ Dysfunction

Myocardial injury in sepsis is intricately intertwined with multi-organ failure, with SCM serving as a critical contributor. Sepsis-induced myocardial dysfunction, characterized by a reversible decline in myocardial contractility, left ventricular cavity dilation, and reduced LVEF, is closely associated with cardiac dysfunction, multiple organ failure, and heightened mortality rates among septic patients [6,48-49]. The pathophysiology of SCM encompasses various mechanisms, including impaired myocardial circulation, direct myocardial depression, and mitochondrial dysfunction [6,48]. This condition significantly exacerbates sepsis-induced cardiac dysfunction and is linked to substantially elevated mortality rates [48]. Managing SCM poses a considerable challenge, with current treatment strategies primarily centered on infection control, mitigating end-organ damage, and providing hemodynamic support [48]. Further research is imperative to deepen our understanding of the pathophysiology of SCM and devise more efficacious therapeutic approaches [6,48].

Role of Myocardial Injury as a Prognostic Marker for Multi-organ Failure

Myocardial injury serves as a crucial prognostic indicator for the development of multi-organ failure. In the context of acute heart failure, the presence of dysfunction or injury in multiple end-organs, including the heart itself, signifies patients at the highest risk of adverse outcomes, with worsening heart failure occurring more frequently in this subset [50]. In severe cases of COVID-19, the ominous prognosis associated with myocardial injury primarily stems from the involvement of multiple organ systems and critical illness [51]. Furthermore, SIMI is linked to heightened mortality rates among septic patients and represents the most prevalent complication of organ dysfunction in this population [49,52]. Research has consistently demonstrated a significant association between myocardial injury and elevated risks of both long-term mortality and in-hospital mortality among individuals with COVID-19, underscoring its pivotal role as a prognostic marker for multi-organ failure [53].

Implications for Patient Management and Prognosis

The implications for patient management and prognosis in SCM are substantial. This condition, characterized by a reversible decline in myocardial contractility, left ventricular cavity dilation, and reduced LVEF, can precipitate cardiac dysfunction, multiple organ failure, and heightened mortality rates among septic patients [54-56]. While the prognostic significance of septic left ventricular dysfunction remains a topic of debate, its treatment remains a subject of ongoing discussion [8]. Presently, management primarily revolves around supportive measures, concentrating on infection control, mitigating end-organ damage, and providing hemodynamic support [54]. Therapeutic interventions aimed at bolstering cardiac function, such as heart rate reduction utilizing agents like ivabradine and beta-blockers, have been posited as potentially beneficial, albeit their utilization remains experimental [56]. The pathophysiology of SCM involves a multitude of mechanisms, including impaired myocardial circulation, direct myocardial depression, and mitochondrial dysfunction [5]. Further research endeavors are imperative to enhance our understanding of this condition and to devise more effective therapeutic strategies [5].

Therapeutic interventions

Pharmacological Approaches

Several alternative pharmacological approaches have been proposed for managing septic shock beyond traditional amine-based therapies. These include beta-blockers, hormone replacement, levosimendan, and angiotensin II [57]. Melatonin, a hormone renowned for its diverse physiological functions, has emerged as a potential treatment avenue for SCM. Its demonstrated anti-inflammatory, antioxidant, and anti-apoptotic effects suggest promising potential for managing this condition [58]. Inotropic drugs such as levosimendan have demonstrated efficacy in reducing mortality among patients with severe sepsis and septic shock. Levosimendan, functioning as a calcium-sensitizing drug, augments the effects of calcium on myofilaments during contraction [55].

**Figure 1:**
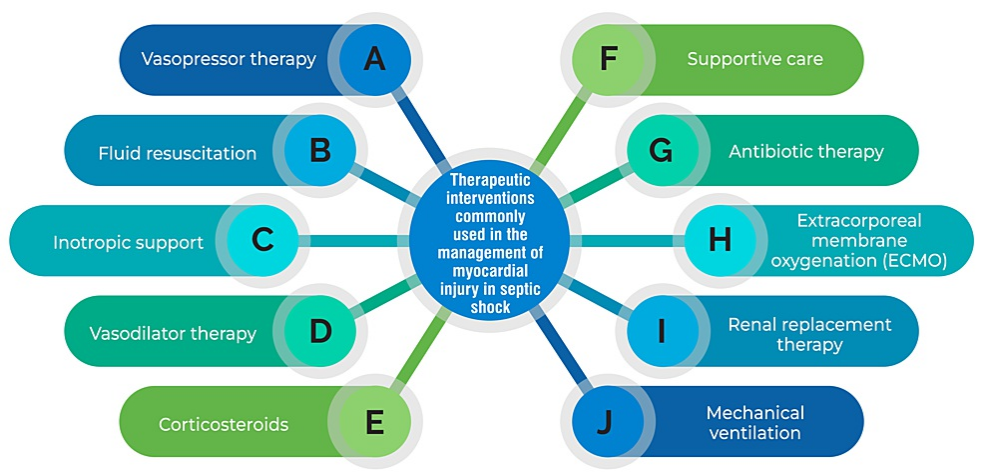
Therapeutic interventions commonly used in the management of myocardial injury in septic shock Image Credit: Corresponding Author

While crystalloids are typically recommended as the initial fluid for resuscitation in sepsis, studies indicate that using albumin and hypertonic saline may offer more significant benefits for cardiac function in septic animals [55]. Norepinephrine stands as the preferred vasopressor for septic patients with low cardiac output, as it enhances cardiac preload and output in individuals facing life-threatening conditions [55]. Furthermore, additional pharmacological approaches have been proposed for managing SCM, including dexmedetomidine, 3,3'-diindolylmethane, and traditional Chinese medicine [8,54-55]. However, it's essential to acknowledge that addressing SCM remains a formidable challenge, emphasizing the pressing need for further research to develop more efficacious therapeutic strategies [55]. Therapeutic interventions commonly used in the management of myocardial injury in septic shock are shown in Figure 1.

Hemodynamic Support

The management of SCM encompasses a range of therapeutic interventions, with hemodynamic support playing a crucial role. While uncertainties persist regarding hemodynamic support for septic shock, initial treatment hinges on prompt recognition, fluid resuscitation, and the administration of vasopressors [8]. The Surviving Sepsis Campaign 2016-2018 strongly advocates norepinephrine as the primary vasopressor choice in septic shock treatment [8]. Furthermore, the utilization of inotropic medications like levosimendan has demonstrated efficacy in reducing mortality rates among patients with severe sepsis and septic shock [56]. Additionally, SCM management has contemplated the potential application of mechanical support devices such as intra-aortic balloon pumps (IABP), percutaneous ventricular assist devices, or ECMO. However, conclusive evidence from randomized controlled trials is lacking [59]. Given the complexity of SCM management, a tailored approach is essential, guided by the individual patient's hemodynamic status and response to treatment.

Targeted Therapies for Myocardial Protection

Targeted therapies aimed at myocardial protection are currently a focal point of research efforts. Among the potential strategies under investigation are heart rate reduction using agents like ivabradine and beta-blockers and inotropic support provided by medications such as levosimendan. Additionally, fluid resuscitation employing albumin and hypertonic saline, along with vasopressor support utilizing norepinephrine, constitute vital components of targeted therapy approaches. Comprehensive supportive care and vigilant monitoring complement these interventions. Moreover, nanocarrier-based targeted therapies, including antibody-modified liposomes, have exhibited promising results in preclinical studies for treating myocardial infarction [60]. Other potential avenues for targeted therapy encompass protein drugs, gene editing technologies, nucleic acid drugs, and cell therapy [61]. However, it is imperative to emphasize the necessity for further research endeavors to refine and optimize these therapeutic modalities for eventual clinical application.

## Conclusions

This comprehensive review has underscored the critical role of myocardial injury in septic shock, emphasizing its intricate interplay with multi-organ failure and its prognostic significance. This review has highlighted the importance of early recognition and monitoring of cardiac biomarkers in guiding clinical management by elucidating the mechanisms underlying myocardial injury, such as inflammation, oxidative stress, and microcirculatory dysfunction. Moreover, the review has underscored the potential therapeutic implications of targeting myocardial injury, ranging from anti-inflammatory agents to supportive measures, in mitigating adverse outcomes associated with septic shock. Future research endeavors should focus on further elucidating the underlying pathophysiological mechanisms of myocardial injury, identifying novel therapeutic targets, and advancing personalized medicine approaches to optimize patient care. Through multidisciplinary collaboration and a deeper understanding of myocardial injury in septic shock, clinicians and researchers can strive towards improving patient outcomes and reducing the burden of multi-organ dysfunction in this challenging clinical scenario.

## Figures and Tables

**Figure 2:**
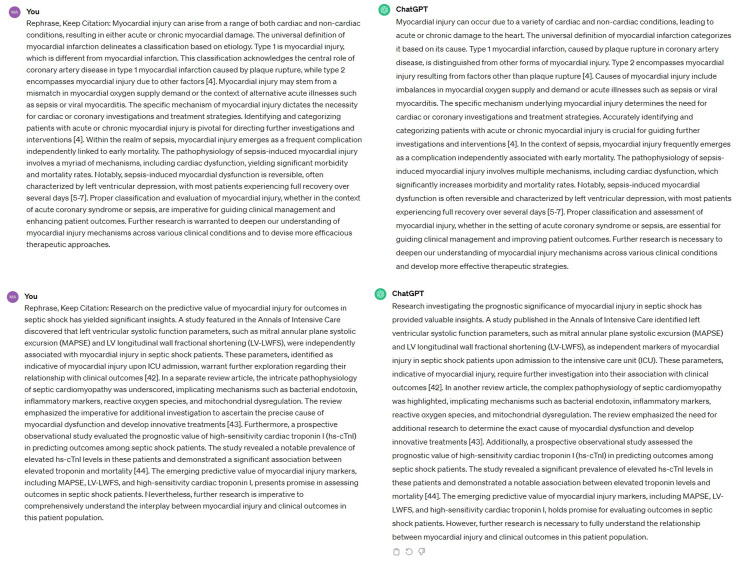
ChatGPT-assisted editing and rephrasing of the review article

## Appendices

The ChatGPT language model (OpenAI, San Francisco, California) was employed to assist in the formulation of key arguments, structuring the content, and refining the language of our manuscript. It provided valuable insights and suggestions throughout the writing process, enhancing the overall coherence and clarity of the article. It was also utilized to assist in editing and rephrasing the work to ensure coherence and clarity in conveying the findings (Figure 2).
